# Development and psychometric evaluation of the trauma nurse core competency scale

**DOI:** 10.3389/fpubh.2022.959176

**Published:** 2022-11-29

**Authors:** Lu Wang, Xinping Zhang, Ping Zhang, Qian Zhou, Qianning Wang, Jing Cheng

**Affiliations:** ^1^Department of Emergency, Tongji Hospital, Tongji Medical College, Huazhong University of Science and Technology, Wuhan, China; ^2^School of Medicine and Health Management, Tongji Medical College, Huazhong University of Science and Technology, Wuhan, China; ^3^Department of Nursing, Tongji Hospital, Tongji Medical College, Huazhong University of Science and Technology, Wuhan, China

**Keywords:** trauma nursing, core competence, scale development, psychometric evaluation, reliability, validity

## Abstract

**Background:**

Trauma, especially severe trauma, has become a significant public health problem worldwide. This postulates higher requirements on the core competence of trauma nurses. However, limited scales exist to assess it validly and reliably. This study aims to develop and evaluate the psychometric properties of the Trauma Nurse Core Competency Scale (TNCCS).

**Methods:**

This study included three stages. First, scale development was based on a broad literature review and two rounds of Delphi expert consultation. Then, a pre-investigation was conducted with 106 trauma nurses, and a formal scale was formed. Finally, scale evaluation of reliability and validity, based on a cross-sectional study, was tested with 1,107 trauma nurses. Content validity and structure validity were used to evaluate the validity of TNCCS. The Cronbach's α coefficient and the split-half reliability coefficient were used to evaluate the reliability of TNCCS.

**Results:**

The final scale contained 46 items under three dimensions, which were Knowledge and skills (21 items), Comprehensive literacy (20 items), and Professionalism & physical and mental health (5 items). The Content Validity Index (CVI) of the total scale was 0.980. The goodness-of-fit indices (χ^2^/df = 3.547, RMSEA = 0.065, GFI = 0.929, CFI = 0.912, NFI = 0.904, IFI = 0.929) signified a good fit for this model. The Construct Reliability (CR) ranged from 0.89 to 0.98, and the Average Variance Extracted (AVE) ranged from 0.62 to 0.69. The Cronbach's α coefficient of the scale was 0.99, ranging from 0.90 to 0.98 for the subscales. The split-half reliability coefficient was 0.84.

**Conclusions:**

The TNCCS demonstrated good validity and reliability, and it could be used to assess the core competency of trauma nurses. The present study has valuable implications for nursing managers to take corresponding measures to train and improve the core competence of trauma nurses.

## Introduction

Trauma is the leading cause of death for people under 45 years, the fourth cause of death from all diseases, and a major contributor to the global burden of disease (1). Centers for Disease Control and Prevention (CDC) reported that trauma injury accounted for 25–33% of unintentional deaths and led to 5.7 million annual deaths globally, and most of them were teenagers, representing a significant impact on productivity and a great economic burden on both the family and society (1–4). In China, the incidence of trauma has been increasing year by year, and it has become the fifth cause of death for urban and rural residents; hence, China is suffering from a heavy burden of patients with trauma (5). Nowadays, trauma and its consequences have been identified as high-priority public health risks (6).

The huge burden brought by trauma puts forward high requirements for the quality of trauma care service. Trauma nursing is a multidimensional specialty area in nursing, and it encompasses a large variety of nursing specialties, such as injury prevention, emergency, perioperative nursing, intensive care, and rehabilitation (7). Trauma nursing involves a wide range of fields, complex work, and high risks, especially for critical trauma patients characterized by an unpredictable disease progression, an unstable physiological status, and a high risk of adverse outcomes (8). Therefore, improving the quality of trauma nursing service requires a high demand on the core competence of trauma nurses.

WHO and the Lancet Commission on Global Surgery jointly assert that capacity building of trauma care providers was essential to address the global burden of trauma (9). The core competency of trauma nurses is critical to ensure patient safety (7), improve the quality of trauma service in medical institutions (7), enhance the psychological resilience of trauma nurses, and avoid vicarious trauma (2). It was confirmed that trauma patients significantly showed lower mortality or morbidity when treated by trauma health professionals with high levels of core competence, especially trauma nurses (2, 7, 10). In long-term care, patients with higher levels of trauma care were more likely to be discharged home, had fewer adverse events, had higher functional status, and the shorter length of stay (11, 12). Conversely, low quality or lack of trauma care skills can reduce the quality and trust of trauma services in healthcare facilities. As the largest group of trauma care, nurses are not immune to the deleterious adverse effects such as post-traumatic stress disorder (PTSD) and psychiatric disorders that are associated with the consequences of trauma (2). In addition, nurses are also exposed to vicarious trauma (13) or compassion fatigue, which refers to the significant emotional and physical toll that takes place when trauma nurses are unable to refuel after caring for those with trauma (14).

Assessment of competence is an essential prerequisite for improving competence (15). Therefore, how to evaluate the core competence of trauma nurses is a critical issue at present. This not only helps to understand the status quo of the core competence level of trauma nurses but also provides valuable evidence-based suggestions for the intervention and effect evaluation in the later period (16).

Establishing a unified, national, and shared standard of competence for nurses is crucial to evaluating nurses' core competencies (17, 18). However, there is no uniform definition of the core competence of trauma nurses at present. From a “competence” perspective, it is identified that “knowledge, skills and attitudes” were the competencies that trauma nurses must possess in 2013 by the “Advancing the Science of Education, Training and Practice in Trauma” national consensus conference on trauma competencies (6). Both foundational (e.g., scientific Knowledge, individual and cultural diversity, and ethical, and legal issues) and functional competencies (e.g., assessment, intervention, evaluation, etc.) were included in the Guideline on Trauma Competencies for Education and Training, also known as the New Haven Trauma Competency model (17). On this basis, the National Major Trauma Nursing Group (NMTNG) developed a framework of trauma care competency from organizational aspects, clinical and technical skills, and non-technical skills (18). Although these guidelines did not clearly define the content of core competence and requirements of trauma nurses, they provided a competence framework and model for the development of core competence assessment tools for trauma nurses (6, 17, 18).

It can be seen from the literature review that the improvement of the core competence of trauma nurses has a positive effect on improving the quality of trauma service, the prognosis of trauma patients, and the physical and mental health of trauma nurses themselves. However, there is currently no reliable and valid tool for evaluating the core competence of trauma nurses. Additionally, there is also no mature theoretical framework or model to guide the construction of the instrument, which is very significant for clinical practice and teaching training. Therefore, this study aims to develop a reliable and valid scale for evaluating the core competence of trauma nurses in China. We propose a hypothesis that the Trauma nurse Core Competency Scale has satisfactory psychometric properties.

## Methods

Scale development and psychometric evaluation research were adopted as this study's design. It covered three phases of scale development, pre-investigation, and scale evaluation. Scale development was based on a board literature review and the Delphi method. Scale evaluation was based on a cross-sectional survey.

### Scale development

#### Conceptualization

The initial step of developing a new scale is to define the concept to be measured. According to the New Haven Trauma Competency model, the core competence of trauma nurses was an integration of knowledge, skills, and attitudes, covering both foundational (e.g., scientific knowledge, individual and cultural diversity, and ethical and legal issues) and functional competencies (e.g., assessment and intervention) specific to trauma field (17). On this basis, the National Major Trauma Nursing Group (NMTNG) developed a framework of trauma care competency from the perspective of organizational aspects, clinical and technical skills, and non-technical skills (18). Besides, these are also indispensable to ensure the safety of trauma patients and improve the quality of trauma care, including a passion for a trauma care career, possession of trauma professionalism, and the resilience to deal with traumatic emergencies and major events, teamwork, leadership, risk management, education and guidance, critical thinking, and personal development capabilities (17, 19, 20). Based on the above, the core competence of trauma nurses in this study is defined as passion, professionalism, psychological quality, professional knowledge and skills, and other comprehensive literacy required to engage in trauma care, such as teamwork, leadership, risk management, education and guidance, critical thinking, and personal development.

### Item pool development

The item pool was derived from three sources: (1) a broad literature review; and (2) two rounds of Delphi expert consultation.

First, Google Scholar, Web of Science, PubMed, Elsevier, EBSCO, CNKI, WAN FANG MEDONLINE, and other websites were searched. Search keywords were “competency, core competency,” “evaluation, evaluation indicators,” and “nurse, trauma, nursing.” We mainly referred to The New Haven Trauma Competency model (17), the Framework of Trauma Care Competence developed by the National Major Trauma Nursing Group (NMTNG) (18), and the Trauma Nursing Core Competence Scale for Vietnamese trauma nurses developed by the Professor Vu DV (7). All items were expressed positively. That means the higher the score, the higher the trauma nurse's level of core competence. A 5-point Likert scale was used to rate the answers, with options ranging from 1 = “incompetent” to 5 = “very competent.” Brislin's back translation was used to ensure the validity of all English sentences (21). Through several rounds of the panel discussion, 8 dimensions and 68 items were determined (Appendix 1).

Then, we invited 12 experts to evaluate the relevance and clarity of all the items. The expert consultation form included experts' information, judgment basis, and the importance level of each item. A 5-point Likert scale was used to rate the items with options ranging from 1 = “not important” to 5 = “important.” The modify column, delete column, and add column have also been added. We asked the experts to return the questionnaire within a week. The evaluation time of each expert varied from 20 to 40 min, with an average of 30 min.

The expert inclusion criteria are as follows: working in a tertiary hospital; bachelor's degree or above; intermediate or above title; engaged in trauma nursing for more than 10 years (range: 10–35; median: 22); and served as the nurse manager of emergency or trauma surgery department.

There were seven masters, five undergraduates, nine women, and three men, and the age ranged from 30 to 58 years (median: 45). The positive coefficient of experts was 100%, and the degree of authority of experts was 0.88. The expert coordination coefficient (W) was 0.21 (*p* < 0.001) and 0.23 (*p* < 0.001), respectively. After each round of consultation, items were edited according to the critical value of the mean, coefficient of variation, and full score ratio. After two rounds of consultation, experts' opinions tend to be consistent. Based on the results of the first round of consultation, eight items were deleted because concepts were too large to measure (Items: 6, 7, 18, 24, 35, 57, 61, 67). Six items were deleted for under importance (Items: 22, 23, 48, 62, 64, 66). We combined items 20 and 36; items 51 and 52; items 53 and 54; and items 55 and 56 due to the repetitive connotations. Additionally, the dimensions remained. In the second round of consultation, we deleted item 40 because it was identical to item 41. Items 34 and 37 were deleted because the content they expressed was contained in item 38. Finally, there were only two items left under the dimension of “risk management capacity,” which should be deleted in principle. However, considering their professional significance, these two items were retained. Hence, the revised questionnaire contained 8 dimensions and 46 items.

### Pre-investigation

First, we distributed 30 questionnaires to trauma nurses in the trauma surgery department of a comprehensive medical institution in China. We asked them to answer these questions: (a) which items they had difficulty responding to and why, (b) which items they had a question about, (c) revisions they believed should be made, and (d) suggestions for items that should be included. While the nurses had no problems in responding to the items, they did suggest some modifications for clarity. For example, modify the expression “Develop multidisciplinary team collaboration to make patient hospitalization and discharge care plans” of item 41 to “work with a multidisciplinary team to discuss and develop a patient hospitalization care plan” because the contents before modification were repeated with Items 42 and 43.

Then, the questionnaire link was sent to the nurse manager of the emergency department and trauma surgery department of the four hospitals, which were the initiators and pioneers of the Chinese Trauma Care Alliance (CTCA), using an online survey. The four hospitals were located in the north, west, central, and east of China, respectively. They all belong to large-scale medical institutions integrating clinical practice, teaching, and scientific research. Besides, the department of trauma surgery of them developed well. The nurse managers would send the questionnaire link to the Wechat group of their department and inform trauma nurses to fill in the questionnaire voluntarily.

A total of 106 valid questionnaires were collected. Appendix 2 gave the demographic characteristics of the 106 nurses. Item-total correlations, critical ratio method, Cronbach's α coefficient, and EFA were adopted to filter and correct items (22, 23). Item-total correlations for the initial 60-item scale ranged from 0.41 to 0.82. The critical ratio of all items differed significantly (*p* < 0.01). The Cronbach's α coefficient of the total scale was 0.91, and the three factors ranged from 0.90 to 0.95. The result (KMO = 0.944, Bartlett's test value = 4,898.860, *P* < 0.001) indicated perfect appropriateness to conduct exploratory factor analysis. According to the results of factor analysis, the dimensions C (Risk management capacity), D (Communication and coordination capacity), E (leadership), F (Education and guidance capacity), G (Critical thinking), and H (The capacity of scientific research and personal development) were rotated to a common factor. According to the literature (18, 23, 24) and the conception, our study named this dimension “Comprehensive Literacy”. According to the above results, the adjusted scale contained 3 dimensions and 46 items.

### Scale evaluation

#### Design, setting, and participants

An online survey was conducted among trauma nurses from four medical institutions (Peking University People's Hospital, Army Medical University, Tongji Hospital, Tongji Medical College, Huazhong University of Science and Technology, and The Second Affiliated Hospital of Zhejiang University), in October 2021, through a directional convenient sampling method. The four medical institutions, which were affiliated with the CTCA, were located in Beijing, Chongqing, Wuhan, and Zhejiang and covered the north, west, central, and east of China. They were the four medical institutions with the highest level of trauma treatment in China. Inclusion criteria: nurses working in comprehensive medical institutions; engaging in the department related to trauma care such as emergency, ICU, trauma surgery, orthopedic trauma, neurosurgery, and operating room; registered nurse; informed and voluntary participation in the study.

### Data collection procedure

The survey was conducted in October and November 2021. We hired a project manager at each hospital, primarily the head nurse of Trauma Surgery, to coordinate and guide the investigation. The researcher was responsible for contacting and training the project managers from each hospital. The managers were in charge of sending the link of the electronic questionnaire to the departments related to trauma care, such as emergency, ICU, trauma surgery, orthopedic trauma, neurosurgery, and operating room. In addition, they were in charge of explaining the research purpose and content to respondents. The purpose of the study was to construct the Trauma Nurse Core Competency Scale in China and then provide evidence for the core competence training of trauma nurses. Respondents were required to fill in the questionnaire according to their actual condition. Whether to fill in the questionnaire was voluntary.

To ensure the quality of data, we adopted the double-check method according to the following requirements: all items were set as “required questions” to guarantee the completeness of the questionnaire; users from each IP address only had one chance to participate in the investigation; the answer time was longer than 2 min.

### Instrument

The formal questionnaire consisted of two parts. The first part was the general information of the respondents, including gender, age (years), educational level, working years of trauma-related departments (years), and whether they participated in trauma care training. The second part was the revised TNCCS, which included 46 items under three dimensions. Each item was designed with a 5-level Likert scale, and the options were “not competent,” “Somewhat competent,” “fairly competent,” “sufficiently competent,” and “very competent.”

### Statistical analysis

The reliability and validity of 46 items were tested. Validity refers to the extent to which the instrument can measure objects, including content validity and structure validity. Content validity was tested by the Content Validity Index (CVI). Structure validity was tested by item analysis, Exploratory Factor Analysis (EFA), and Confirmatory Factor Analysis (CFA). Reliability refers to the consistency and stability of test results, which was tested by Cronbach's α internal consistency coefficient and split-half reliability coefficient. The total score of the scale was the sum of the scores of each item. The IBM SPSS 22.0 statistics software and IBM SPSS Amos 24.0 software (SPSS Inc., IBM Company, Chicago, IL) were used to manage and analyze the dataset. For all analyses, *p* < 0.05 was considered statistically significant.

### Content validity

The content validity of the scale was measured by CVI and the item relevance scoring method was based on expert consultation. Experts were invited to rate items on a scale of 1–4 (1 being irrelevant, 2 being relevant, 3 being fairly relevant, and 4 being highly relevant) on their relevance to the dimensions they belong to. Item-level CVI (I-CVI) was the number of experts giving a 3- or 4-point evaluation for each item divided by the total number of experts and is generally expected to exceed 0.78 (24). For scale-level CVI (S-CVI), the scale-level content validity/universal agreement (S-CVI/UA) should be >0.8, and the scale-level content validity index/average (S-CVI/AVE) should be >0.9 to reach an ideal level (25).

### Construct validity

Item analysis, EFA, and CFA were used to test the validity of the structure. If the Spearman correlations of the factor-total score ranged from 0.3 to 0.8, the factor intercorrelations were <0.8, and the Spearman correlations of the item-total score were between 0.3 and 0.8.

The total score of the questionnaire was ranked, with the top 27% (≥184 points) as the higher group, and the last 27% (≤130 points) as the lower group (26). If the independent *t*-test showed that the Critical Ratio (CR) of each item differed significantly between the higher-score group and the lower-score group, we could infer that the scale had good homogeneity and discrimination (26).

Exploratory Factor Analysis was mainly used to re-evaluate and filter items. We performed the Bartlett test of sphericity for all the items and calculated the Kaiser–Meyer–Olkin (KMO) index. The Bartlett test of sphericity was significant (*p* < 0.05), and KMO scores >0.7 were considered appropriate for factor analysis (27). Principal component analysis (PCA) with Varimax rotation was adopted. Generally, each factor has at least three items, and the items would be retained unless their factor loading >0.5, commonality >0.2, eigenvalue >1, and deviation of factor loading between different factors <0.2 (28, 29).

Based on the EFA screening items, CFA evaluates the entire model and filters the problematic items again. Overall, model evaluation: χ^2^/df, root mean square error of approximation (RMSEA), goodness-of-fit index (GFI), comparative fit index (CFI), Normed Fit Index (NFI), Incremental Fit Index (IFI) were used to evaluate the model fit. In theory, if χ^2^/df <3, RMSEA <0.08, GFI > 0.90, CFI > 0.90, NFI > 0.90, IFI > 0.90, then it indicates that the goodness-of-fit index is reasonable and acceptable (30). But in fact, according to relevant studies, χ^2^/df <5 is also acceptable (31, 32). Measurement model evaluation: The convergent validity and discriminant validity would be regarded as enough if the following standards were achieved: (1) standardized regression coefficients of each item for latent variables > 0.6; (2) construct reliability was more than 0.6; (3) average variance extracted (AVE) was >0.5; (4) the square root of the average variance extracted exceeded the correlation coefficient (33). In CFA, entries that do not meet the measurement model criteria will be deleted. When the overall fit model is insufficient, the model modification index is used to modify the model. To ensure the independence of latent variables, only the correlation of item residuals in the same dimension is added.

### Reliability

Cronbach's α coefficients of the whole scale and each dimension were calculated to assess the internal consistency of the scale. Cronbach's α coefficients ranged from 0 to 1. The larger the value of Cronbach's α coefficients, the better the consistency. Generally, a value >0.7 was considered good consistency (34). The split-half reliability coefficient is considered to be another indicator of reliability. When calculating it, we divided items into two groups based on parity. Then, the correlation coefficient between the two groups was calculated, and the Spearman-Brown formula was applied to estimate the reliability of the whole scale. It was agreed that the split-half reliability coefficient should be >0.7 to reach an acceptable level.

### Ethical considerations

This study was approved by the Tongji Hospital, Tongji Medical College, and Huazhong University of Science and Technology (TJ-IRB20210920). All the surveys were kept confidential and anonymous.

## Results

### Characteristics of participants

A total of 1,203 nurses participated in the online survey, whereas 96 samples were removed because the answers to all 46 items in a questionnaire were the same or the answer time was <2 min. Finally, 1,107 nurses were included in the study, with a 92% effective response rate. The samples were divided into the first 474 cases (Sample 1) and the last 613 cases (Sample 2) according to the recovery time. Sample 1 was used for EFA and sample 2 was used for CFA. The demographic characteristics of participants were shown in Table 1.

**Table 1:** The demographic characteristics of participants.

**Characteristics**	**Sample A (*****n*** = **494)**		**Sample B (*****n*** = **613)**	
	***N***	**%**	***N***	**%**
**Gender**
Male	38	7.69	49	7.99
Female	456	92.31	563	91.84
**Age (years)**
≤25	75	15.18	89	14.52
26–35	294	59.51	301	49.10
36–45	84	17.00	167	27.24
>45	41	8.31	56	9.14
**Educational level**
Junior college	113	22.87	187	30.51
Bachelor's degree	286	57.89	321	52.37
Master's degree or above	95	19.23	105	17.13
**Working years of trauma-related departments (years)**
<1	118	23.89	101	16.48
1–3	108	21.86	189	30.83
4–6	72	14.57	101	16.48
7–9	63	12.75	89	14.52
≥10	133	26.92	133	21.70
**Have participated in trauma care training**
Yes	435	88.06	571	93.15
No	59	11.94	42	6.85

### Validity

#### Content validity

The experts invited were the same in the second round of consultation. For I-CVI, they ranged from 0.83 to 1. For S-CVI, S-CVI was 0.98; S-CVI/UA was 0.89 (>0.8); and S-CVI/AVE was 0.95 (>0.9). The content validity of the scale was good.

#### Construct validity

##### Item analysis

Item-total correlations for the 46 items ranged from 0.46 to 0.87, as shown in Table 2. The correlation between factors and the factor-total score was 0.54–0.81 and 0.64–0.95, respectively (**Table 4**). The mean inter-item correlations within the scale ranged from 0.71 to 0.73 (Table 2). All of the items were retained. In addition, the independent *t*-test showed that the Critical Ratio (CR) of each item differed significantly (*p* ≤ 0.01) between the higher-score group (upper 75%, score ≥ 184) and the lower-score group (under 25%, score ≤ 130). Thus, the 46 items had good homogeneity and discrimination and were retained to test construct validity.

**Table 2:** Psychometric assessment and construction validity.

**Dimensions and items**	**Factors**			**Critical ratio (*t*)**	**ITC**	**Commonality**	**Cronbach's α**	**MIIC**
					**Coefficient**			
	**1**	**2**	**3**				**0.99**	
**Dimension 1 Knowledge and skills**							0.98	0.71
28. Trauma treatment process and nursing cooperation	**0.82**	0.35	0.17	26.15^**^	0.84	0.83		
13. Emergency measures and nursing care of trauma hemostatic	**0.82**	0.29	0.22	23.95^**^	0.81	0.80		
27. Trauma patient transport technique	**0.81**	0.36	0.16	27.46^**^	0.83	0.82		
29. Airway management and respiratory support	**0.81**	0.35	0.13	25.37^**^	0.81	0.81		
15. Care of trauma complications	**0.80**	0.34	0.23	25.55^**^	0.84	0.83		
12. Observation and nursing of multiple injuries	**0.79**	0.32	0.23	23.20^**^	0.82	0.79		
26. On-site first aid technique for trauma	**0.79**	0.39	0.15	28.13^**^	0.83	0.79		
25. Injury assessment and triage	**0.77**	0.36	0.14	26.08^**^	0.81	0.75		
10. Traumatic shock assessment monitoring and resuscitation	**0.77**	0.26	0.20	20.48^**^	0.76	0.70		
33. Methods of using assistive tools for trauma patients	**0.75**	0.42	0.12	26.23^**^	0.82	0.75		
9. Trauma assessment methods	**0.74**	0.29	0.18	22.76^**^	0.76	0.70		
14. Management of nursing care before emergency trauma surgery	**0.73**	0.33	0.23	22.45^**^	0.79	0.70		
11. Evaluation and treatment technology of traumatic triad (hypothermia, acidosis and coagulation disorders)	**0.73**	0.33	0.26	24.41^**^	0.80	0.72		
16. Prevention and control of trauma infection	**0.73**	0.42	0.19	28.24^**^	0.84	0.80		
32. Pipeline observation and nursing	**0.72**	0.39	0.19	26.23^**^	0.80	0.74		
30. Establishment of venous access and fluid resuscitation	**0.71**	0.35	0.22	19.98^**^	0.76	0.74		
17. Assessment and management of post-traumatic pain	**0.70**	0.42	0.23	25.24^**^	0.83	0.76		
8. Injury mechanism	**0.67**	0.37	0.18	23.63^**^	0.76	0.67		
31. Emergency trauma surgery nursing cooperation	**0.62**	0.44	0.14	20.24^**^	0.75	0.61		
19. Burn and various wound care	**0.61**	0.41	0.16	25.99^**^	0.76	0.69		
21. Trauma rehabilitation technique	**0.60**	0.43	0.09	22.76^**^	0.72	0.73		
**Dimension 2 Comprehensive literacy**							0.98	0.73
59. Using critical thinking to analyze trauma data to improve the quality of trauma care	0.36	**0.81**	0.15	28.75^**^	0.84	0.82		
58. Provide personalized and differentiated care for trauma patients	0.38	**0.79**	0.24	35.79^**^	0.87	0.84		
60. Application of nursing procedures to provide care for trauma patients and their families	0.37	**0.79**	0.20	29.84^**^	0.84	0.84		
55. Understand the learning needs of the trainee and give the standard guidance	0.36	**0.78**	0.17	27.29^**^	0.81	0.77		
44. Actively participate in making department plans	0.30	**0.78**	0.15	27.29^**^	0.77	0.73		
53. Understand the learning needs of junior nurses and give guidance to them	0.38	**0.77**	0.21	31.98^**^	0.84	0.80		
51. Understand the needs of trauma patients and their families, and give professional intervention and guidance	0.38	**0.77**	0.22	28.12^**^	0.84	0.80		
41. Develop multidisciplinary team work to develop patient hospitalization and discharge care plans	0.37	**0.76**	0.13	28.11^**^	0.80	0.74		
50. The Ability to resolve conflict	0.38	**0.76**	0.20	27.34^**^	0.82	0.77		
63. Ability to detect scientific problems	0.26	**0.75**	0.22	22.88^**^	0.75	0.71		
68. Professional self-improvement ability	0.31	**0.74**	0.23	23.58^**^	0.77	0.69		
42. Assist trauma patients in need to transfer to rehabilitation institutions	0.38	**0.73**	0.13	28.40^**^	0.79	0.72		
43. Provide extended care after discharge	0.30	**0.73**	0.10	23.00^**^	0.74	0.71		
49. Team management ability	0.40	**0.72**	0.28	27.73^**^	0.83	0.78		
65. Evidence-based nursing capability	0.28	**0.71**	0.15	20.37^**^	0.72	0.67		
47. The ability to create a collaborative working atmosphere	0.37	**0.70**	0.33	25.38^**^	0.81	0.80		
39. The ability to deal with trauma nurse-patient disputes	0.48	**0.70**	0.15	31.80^**^	0.84	0.76		
38. Ability to prevent potential safety hazards of trauma patients	0.53	**0.68**	0.18	33.52^**^	0.87	0.79		
46. Accomplish tasks in an organized and planned way	0.42	**0.68**	0.35	28.56^**^	0.83	0.82		
45. Prioritize tasks in urgent order	0.41	**0.67**	0.36	27.37^**^	0.82	0.80		
**Dimension 3 Professionalism & physical and mental health**							0.90	0.71
4. Professionalism such as active service and self-reliance	0.18	0.25	**0.82**	12.35^**^	0.55	0.77		
5. Good physical and mental quality	0.21	0.22	**0.80**	12.23^**^	0.55	0.73		
1. Observe the laws and regulations	0.15	0.17	**0.78**	9.44^**^	0.46	0.67		
3. Passionate about trauma care	0.30	0.22	**0.76**	13.91^**^	0.60	0.74		
2. Observe ethical principles	0.23	0.22	**0.76**	11.26^**^	0.55	0.68		

Extraction method: principal component analysis; rotation method: Varimax. ITC, item-total correlation; MIIC, mean inter-item correlation.

** p < 0.01. The bold values were factor loadings rotated to a common factor.

##### Exploratory factor analysis

The results of EFA showed that Bartlett's test of sphericity was significant (*p* < 0.001) and Kaiser–Meyer–Olkin (KMO) test was 0.91, indicating a high correlation among items, and thus a factor analysis could be performed (*p* < 0.001). The results of the factor analysis were shown in Table 3. Totally three components were retrieved, which together accounted for 75.24% variance and explained with eigenvalue from 1.20 to 30.99 (Shown in Table 3). The commonality among the 46 items was between 0.61 and 0.84. The factor loadings were from 0.56 to 0.82.

**Table 3:** Selected analysis results using varimax rotation and principal component extraction.

**Component**	**Initial Eigenvalue**		
	**Total**	**% of Variance**	**Cumulative %**
1	30.99	67.36	67.36
2	2.42	5.27	72.63
3	1.20	2.62	75.24

##### Confirmatory factor analysis

**Figure 1:**
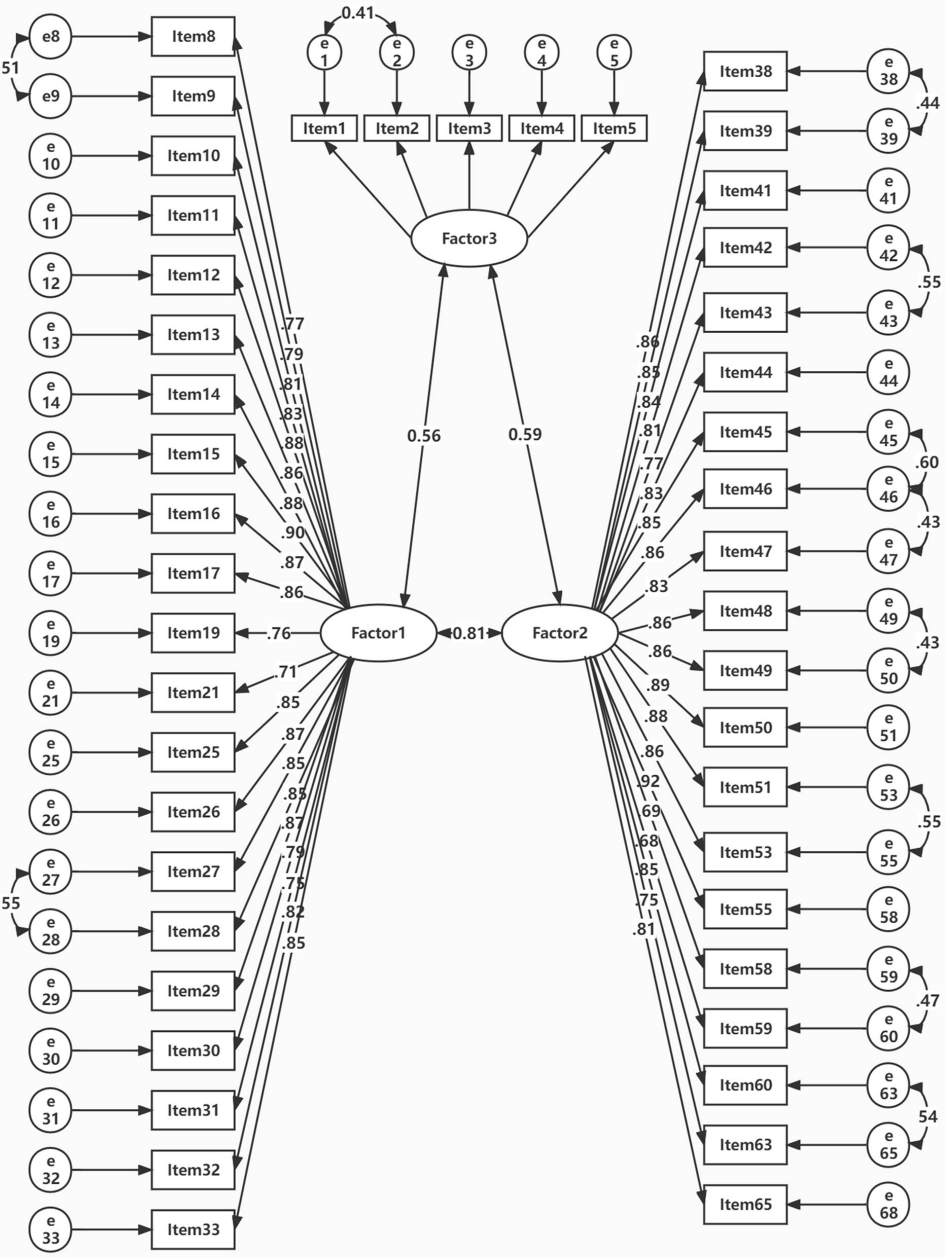
Standardized parameter estimates for the second-order TNCCS.

The maximum likelihood estimation method (MLE) suggested that the fitting indexes of the three-factor model reached the reference value after adjustment and the model fit well (χ^2^/df = 3.57, RMSEA = 0.065, GFI = 0.929, CFI = 0.912, NFI = 0.904, IFI = 0.929). The range of standardized regression coefficients of each item for latent variables for three factors was separately 0.715–0.894, 0.7–0.9, and 0.698–0.896 (shown in Figure 1). The CR ranged from 0.89 to 0.98, and the AVE ranged from 0.62 to 0.69. The square root of the average variance extracted were shown in Table 4.

**Table 4:** The correlation coefficient between factors and factor-total score, the square root of the average variance extracted.

	**Factor 1**	**Factor 2**	**Factor 3**	**Total score**
Factor 1	0.87	0.81	0.54	0.95
Factor 2		0.89	0.56	0.95
Factor 3			0.73	0.64

### Reliability

Regarding the 46 items and three factors, Cronbach's α coefficient of the total scale was 0.99, and the three factors ranged from 0.90 to 0.98, all >0.70, which demonstrated that the scale had good internal consistency reliability. As for the split-half reliability coefficient, statistics analysis showed the correlation coefficient of the two groups was 0.80, and the split-half reliability coefficient (Spearman-Brown coefficient) was 0.84, which was >0.7, also indicating good internal reliability of the scale.

## Discussion

The core competence of trauma nurses is vital to improving the quality of trauma service. However, there is still a lack of available instruments to evaluate the core competency of trauma nurses in China. Based on the New Haven Trauma Competency model (17) and the framework of trauma care competency (18), our study attempts to develop an instrument to evaluate the core competence of trauma nurses by combining qualitative and quantitative methods. According to the authoritative results of expert consultation and data simulation from CFA, we constructed a tool with good reliability and validity to evaluate the core competence of trauma nurses in China. The final version of the TNCCS contains 46 items under the following three dimensions: knowledge and skills, comprehensive literacy, and professionalism & physical and mental health.

In 2015, Professor Vu developed Trauma Nursing Core Competence Scale for Vietnamese trauma nurses (7). The dimensions included legal and ethical practice, comfort enhancement, cooperation, partnership/guidance, leadership and management, critical thinking for caring, risk management, and clinical practice (7). The content validity and Cronbach's α coefficient reported in this study met the requirements. However, the article did not report a clear theoretical framework and methodological process for the construction of the scale. Therefore, its rigor and science were beyond examination. Moreover, Cronbach's α coefficient and the content validity of the scale in our study showed better reliability and validity. This had vital implications for improving the core competence of trauma nurses and the quality of trauma service.

During the formal investigation, three factors were extracted from the factor analysis, explaining more than 75% of the variance, which was acknowledged as adequate to capture the main features of a phenomenon (22). The Cronbach's α coefficient and the split-half reliability coefficient of the whole scale were above 0.7, indicating that the scale had good reliability. In addition, I-CVI and S-CVI/AVE were above 0.8, which showed that they had good content validity. In terms of construct validity, the results of the item analysis all met the requirements. Besides, as shown by χ^2^/df, RMSEA, GFI, CFI, NFI, and IFI, the result of CFA fitted the model well, indicating good construct validity.

The first factor was knowledge and skills, mainly theoretical Knowledge and clinical skills that trauma nurses need to master, such as trauma assessment, emergency treatment, transportation, and trauma rehabilitation. This was consistent with the requirements of the Nursing Core Competency Framework from ICN (35) and the New Haven Trauma Competency model (7). It was emphasized that “the ability to directly provide clinical care” was the foundation of the core competence framework, and also the basis of the core competence of the general registered nurse (35). If there was none or a lack of this ability, it was not a specialist nurse, nor could it be distinguished from those experts engaged in nursing education and nursing management (35). Trauma nursing involves a wide range of fields, complex work content, and high risks. Therefore, trauma nurses need adequate knowledge and skills in all aspects and stages of trauma patient management. For example, army family nurse practitioners need to master all skills identified in the Advanced Trauma Life Support (ATLS), Advanced Trauma Care for Nursing (ATCN), and Trauma Nursing Core Course (TNCC) competency evaluation checklists to better manage trauma patients (36). Besides, the role of Trauma Nurse Practitioners (TNPs) was incorporated into the general function of all Acute Care Nurse Practitioners (ACNP), so they were also required to master theoretical Knowledge and practical skills related to emergency nursing and disaster nursing (37, 38). However, compared with the core competence of emergency nurses (39–41) and disaster nurses (42–45), the core competence of trauma nurses is more specialized, specializing in the service of patients with trauma and focusing on the whole process of trauma treatment (pre-hospital—in-hospital—post-discharge rehabilitation) (6, 7). The core competence requirements of emergency nurses are relatively extensive, mainly serving outpatients (41). The core competence requirements of disaster nurses are more extensive, including the work content of emergency nurses and trauma nurses, but it puts more emphasis on the treatment in a disaster environment and the sorting of batch casualties (42). In all items, the Trauma treatment process and nursing cooperation, Emergency measures, and nursing care of trauma hemostatic had the highest factor loading, indicating that they were the most basic and important part of trauma nursing core competencies (7, 46). This also inspired managers to pay attention to strengthening relevant training in the future.

The second factor was Comprehensive literacy, including Critical thinking, Education and guidance capacity, Leadership, Risk management capacity, Communication and coordination capacity, the capacity of scientific research, and Professional development. These were the comprehensive competencies required to engage in trauma care work (17), and they were also the comprehensive competencies required by general registered nurses in the ICN framework (35). Among these items, “Using critical thinking to analyze trauma data to improve the quality of trauma care” and “Provide personalized and differentiated care for trauma patients” had the highest factor loadings, and both belonged to the sub-dimension of critical thinking, indicating that critical thinking was a most significant ability in comprehensive literacy. Critical thinking was the foundation of clinical decision-making (17). As we all know, the condition of trauma patients, especially those with severe trauma, was complex and dynamic, and nurses' decision-making which was based on the patient's dynamic condition changes, that is, critical thinking decisions, could improve the safety of trauma patients (47). Critical thinking was a very important dimension in Professor Vu's research on the core competence of Trauma nurses in Vietnam (8), and this competence was at a high level in both emergency nurses (40) and disaster nurses (44). Second, the items “Understand the learning needs of the trainee and give the standard guidance” and “Actively participate in making department plans” had higher factor loadings, which represented “Teaching-coaching ability” and “leadership”, respectively. Teaching-coaching described the competencies related to the teaching-learning process for patients, families, and junior nurses. Some previous studies only emphasized patient education and did not include the coaching of other nurses (48, 49). This indicated that coaching was not a traditional expectation of a nurse. It suggested that we should consider how to prepare for the teaching-coaching role of nurses (50). Lack of particular patient teaching skills is a barrier to effective patient education practices (51); similarly, a lack of coaching skills impedes junior nurses' professionalization (50). Leadership was one of the dimensions of trauma nurses' core competencies and was the ability of trauma nurses to perform leadership functions, regardless of their specific position. Items in this dimension reflected nurses' overall leadership and influencing competence (9). This finding suggested a major shift in how Chinese trauma nurses perceived themselves, as nurses had traditionally been considered followers and passive subordinates (52). The leadership of trauma nurses can foster capacity development across the surgical system (9), and also could improve other abilities in comprehensive literacy such as communication and coordination (53). The ability of risk management, communication and coordination, and scientific research and professional development were also indispensable to ensuring the safety of trauma patients and improving the quality of trauma care (17, 19, 20).

The third factor, Professionalism & physical and mental health was the combination of professional quality and personal quality. It included adherence to legal and ethical principles, passion for trauma care, the spirit of active service and self-discipline, and good physical and mental quality. The findings were highly congruent with the ICN framework in the legal/ethical practice dimension, which indicated that with the increasing complexity of healthcare delivery, ensuring the protection of individuals and the community is the critical competency of nurses globally (35). However, few existing competence instruments include this important component as an independent assessment dimension. It suggests that more attention is needed to further validate the behavioral indicators of the legal/ethical practice dimension, which was identified in the current study. Trauma nurses must be highly motivated individuals who are committed to the care and rehabilitation of their patients. A positive outlook and a sense of humor are essential in helping patients to cope with the challenges they face (19). Therefore, passion for a trauma care career and possession of the spirit of active service and self-discipline are also very demanding (17, 19, 20). Trauma nursing is both mentally and physically demanding, so good physical and mental fitness are all necessary for working in trauma care (17, 19, 20).

## Limitations and strengths

To ensure the representativeness of the sample, we have collected questionnaires of trauma nurses from four representative comprehensive medical institutions in different regions (north, east, west, and central China). But the sample is only from one country, so the sample size may be relatively small. In the future, it can be applied in different countries and the difference in results can be compared. Second, due to the impact of COVID-19, we adopted an online survey, which may lead to a low response rate. In the future, a field investigation can be conducted after the epidemic and the results of which can be compared with an online survey to see whether there is any difference. Third, we did not do a re-test reliability because online surveys could not guarantee that the participants of the two surveys were the same person. In the future, a field survey can be conducted to verify test-retest reliability. Fourth, the data were collected in the form of self-reports, so respondents may overestimate or underestimate their competence, which may lead to a decline in data accuracy. In the future, more objective indicators can be constructed to evaluate the core competence of trauma nurses.

The greatest contribution of our study is the identification of the core competency elements of trauma nurses. The TNCCS constructed in this study can not only be used to evaluate the core competency level of trauma nurses but also can provide evidence-based recommendations for subsequent training and evaluation of trauma nurses' core competencies.

## Conclusion

Based on the New Haven Trauma Competency model and the framework of trauma care competency, our study has constructed the TNCCS. The TNCCS comprises 3 dimensions and 46 items and exhibits good validity and reliability. The TNCCS can not only be used to evaluate the level of core competence of trauma nurses but also be used for the construction of relevant courses and subsequent training. Educators and managers of trauma nursing can design relevant curriculum systems according to the core competence elements of trauma nurses (knowledge and skills, comprehensive literacy, and professionalism & physical and mental health), including the content of training courses, training methods, evaluation indicators, and evaluation methods. Trauma nurses can make self-evaluations according to the core competence elements of trauma nursing and improve their self-ability.

## Data availability statement

The raw data supporting the conclusions of this article will be made available by the authors, without undue reservation.

## Ethics statement

The studies involving human participants were reviewed and approved by the Ethics Committee of Tongji Hospital, Tongji Medical College, Huazhong University of Science and Technology (TJ-IRB20210920). The patients/participants provided their written informed consent to participate in this study.

## Author contributions

LW conducted the literature review, took part in the investigation, contributed to the data cleaning, performed formal analysis, and wrote the original draft. JC and XZ designed the study, obtained funding, contributed to the interpretation of the results, and performed revisions of the manuscript. PZ, QZ, and QW took part in the investigation, contributed to the data cleaning, performed formal analysis, and performed revisions of the manuscript. All authors contributed to the article and approved the submitted version.

## Conflict of interest

The authors declare that the research was conducted in the absence of any commercial or financial relationships that could be construed as a potential conflict of interest.

## Publisher's note

All claims expressed in this article are solely those of the authors and do not necessarily represent those of their affiliated organizations, or those of the publisher, the editors and the reviewers. Any product that may be evaluated in this article, or claim that may be made by its manufacturer, is not guaranteed or endorsed by the publisher.
