# Supplementary material for: Development and psychometric evaluation of the trauma nurse core competency scale
Source: Front Public Health. 2022 Nov 29;10:959176. doi: 10.3389/fpubh.2022.959176 (PMC9745320; doi:10.3389/fpubh.2022.959176)
Supplement: Supplementary file 1 [file Data_Sheet_1.doc]

APPENDIX 1 The dimensions and pool of items of the initial scale

| Dimension and items |
| --- |
| A Professionalism and physical and mental health  1. Observe the laws and regulations  2. Observe ethical principles  3. Passionate in trauma care  4. Professionalism such as active service and self-discipline  5. Good physical and mental quality  B Theoretical knowledge and clinical skills  B1 Theoretical knowledge   1. Introduction and New Progress of Trauma Care   7. Trauma prevention and Control Education   1. Traumatic mechanism 2. Trauma assessment methods 3. Traumatic shock assessment monitoring and resuscitation 4. Evaluation and treatment technology of traumatic lethal triad (hypothermia，acidosis and coagulation disorders) 5. Observation and nursing of multiple injuries 6. Emergency nursing measures of trauma hemostasis 7. Preoperative nursing management of emergency trauma surgery 8. Care of trauma complications 9. Prevention and control of trauma infection 10. Assessment and management of post-traumatic pain 11. Traumatic nutrition 12. Burn and various wound care 13. Evaluation and intervention of traumatic psychological disorder 14. Trauma rehabilitation technique   B2 Clinical skills   1. Trauma of special population (children, pregnant women, the elderly) 2. Disaster management 3. Quality control index and nursing measures of trauma nursing 4. Injury assessment and triage 5. On-site first aid technique for trauma 6. Trauma patient transport technique   28. Trauma treatment process and nursing cooperation  29. Airway management and respiratory support  30. Establishment of venous access and fluid resuscitation  31. Emergency trauma surgery nursing cooperation  32. Pipeline observation and nursing  33. Usage of assistive devices for trauma patients  C Comprehensive literacy  C1 Risk management capacity   1. Predict the patient's condition and identify potential complications 2. Implement trauma prevention in accordance with national policies 3. Discover the psychological problems of trauma patients in time and carry out intervention   37. Identify common cognitive disorders and enhance self-protection awareness of trauma patients  38. Capacity to prevent potential safety hazards of trauma patients  39. Capacity to deal with trauma nurse-patient disputes  C2 Communication and coordination capacity   1. Assess patients before admission and discharge 2. Develop multidisciplinary team collaboration to make patient hospitalization and discharge care plans 3. Assist trauma patients in need to transfer to rehabilitation institutions 4. Provide extended care post-discharge   C3 Leadership   1. Actively participate in making department plans 2. Prioritize tasks in urgent order 3. Accomplish tasks in an organized and planned way 4. The capacity to create a collaborative working atmosphere   48. Actively communicate and adopt constructive suggestions  49. Team management capacity  50. Capacity to resolve conflict  C4 Teaching–coaching capacity   1. Understand the needs of trauma patients and their families, and give professional intervention and guidance   52. Provide health education for trauma patients and their families  53. Understand the learning needs of junior nurses and give standard guidance  54. Standardized teaching for junior nurses   1. Understand the learning needs of the trainee and give the standard guidance 2. Teach and guide trainees 3. Provide professional advice   C5 Critical thinking   1. Provide personalized and differentiated care for trauma patients 2. Using critical thinking to analyze trauma data for improving the quality of trauma care 3. Using nursing procedures to provide care for trauma patients and their families 4. Evaluate the impact of trauma care on patient outcomes   H The capacity of scientific research and Professional development   1. Willingness and enthusiasm to participate in scientific research 2. Capacity to detect scientific problems 3. Capacity of literature search 4. Evidence-based nursing capacity   66. Focus on trauma care trends  67. Be aware of the strengths and weaknesses of the profession  68. Professional self-improvement capacity |

APPENDIX 2 The Demographic Characteristics of Participants in Pilot Survey (N = 106).

| Characteristics | Category | N (%) |
| --- | --- | --- |
| Gender | Male | 8 (7.55) |
|  | Female | 98 (92.45) |
| Age (years) | ≤25 | 16 (15.09) |
|  | 26~35 | 57 (53.77) |
|  | 36~45 | 24 (22.64) |
|  | ＞45 | 9 (8.49) |
| Educational level | Junior college | 29 (27.36) |
|  | Bachelor’s degree | 58 (54.72) |
|  | Master’s degree or above | 19 (17.92) |
| Working years of trauma-related departments (years) | ＜1 | 21 (19.81) |
|  | 1-3 | 28 (26.42) |
|  | 4-6 | 17 (16.04) |
|  | 7-9 | 15 (14.15) |
|  | ≥10 | 25 (23.58) |
| Have participated in trauma care training | Yes | 96 (90.57) |
|  | No | 10 (9.43) |
